# Bridging the Gap between Vertebrate Cytogenetics and Genomics with Single-Chromosome Sequencing (ChromSeq)

**DOI:** 10.3390/genes12010124

**Published:** 2021-01-19

**Authors:** Alessio Iannucci, Alexey I. Makunin, Artem P. Lisachov, Claudio Ciofi, Roscoe Stanyon, Marta Svartman, Vladimir A. Trifonov

**Affiliations:** 1 Department of Biology, University of Florence, 50019 Sesto Fiorentino, Italy; claudio.ciofi@unifi.it (C.C.); roscoe.stanyon@unifi.it (R.S.); 2 Wellcome Sanger Institute, Hinxton CB10 1SA, UK; alex.makunin@gmail.com; 3 Institute of Molecular and Cellular Biology SB RAS, 630090 Novosibirsk, Russia; vlad@mcb.nsc.ru; 4 Institute of Environmental and Agricultural Biology (X-BIO), University of Tyumen, 625003 Tyumen, Russia; aplisachev@gmail.com; 5 Institute of Cytology and Genetics SB RAS, 630090 Novosibirsk, Russia; 6 Departamento de Genética, Ecologia e Evolução, Universidade Federal de Minas Gerais, Belo Horizonte 31270-901, Brazil; svartmanm@hotmail.com

**Keywords:** chromosomics, cytogenomics, microdissection, flow sorting, DOPseq, *Anolis*, karyotype, reference genomes, *de novo* assembly

## Abstract

The study of vertebrate genome evolution is currently facing a revolution, brought about by next generation sequencing technologies that allow researchers to produce nearly complete and error-free genome assemblies. Novel approaches however do not always provide a direct link with information on vertebrate genome evolution gained from cytogenetic approaches. It is useful to preserve and link cytogenetic data with novel genomic discoveries. Sequencing of DNA from single isolated chromosomes (ChromSeq) is an elegant approach to determine the chromosome content and assign genome assemblies to chromosomes, thus bridging the gap between cytogenetics and genomics. The aim of this paper is to describe how ChromSeq can support the study of vertebrate genome evolution and how it can help link cytogenetic and genomic data. We show key examples of ChromSeq application in the refinement of vertebrate genome assemblies and in the study of vertebrate chromosome and karyotype evolution. We also provide a general overview of the approach and a concrete example of genome refinement using this method in the species *Anolis carolinensis*.

## 1. Introduction

Reference genomes are crucial to investigate many biological aspects of a species. Rough drafts are often sufficient to provide an overview of genome organization, however, chromosome-level assemblies are essential for a more detailed investigation of evolutionary processes and functional annotation, particularly for complex organisms [1]. For vertebrates, in particular, the availability of high-quality chromosome-level reference genomes has led to significant outcomes in comparative and functional genomics (e.g., [2,3,4,5,6,7]). Moreover, high-quality genome assemblies were extremely valuable for population and conservation genomic studies [8,9]. Indeed, chromosome level assemblies are fast becoming the gold standard for *de novo* whole-genome sequencing [1].

Such levels of accuracy can now be reached thanks to recent advances in genome sequencing technologies that lead to the development of techniques to produce complete, contiguous, phased, and ordered representation of the DNA sequence of chromosomes [10]. Long-read sequencing technologies, implemented by Pacific Biosciences (PacBio) and Oxford Nanopore, provide exceptional improvements in contig sizes of genome assemblies [11,12]. These technologies are usually integrated with additional information to orient and order the contigs, a process known as scaffolding, in order to reach a complete chromosome level. Scaffolding is usually performed using chromatin conformation capture techniques (3C, 4C, 5C, Hi-C, and Omni-C, Ref. [13]; Chicago approach, Ref. [14]) and optical mapping methods (BioNano, Ref. [15]). Linked-read sequencing (10× Chromium, Ref. [16]) is also a valid alternative for scaffolding. These techniques can produce nearly error-free assemblies with a number of scaffolds or group of scaffolds equal to the haploid number of the analyzed species [17]. However, a direct information link is not always provided with the karyotype (i.e., the collection of images of all chromosomes of an organism or species) of the target species. For example, “chromosome 7” of a chromosome-level assembly may not necessarily correspond to “chromosome 7” as described at the cytogenetic level [18]. In fact, in assemblies that miss a comprehensive physical assignment to chromosomes, scaffolds are usually ordered and named based on their size. In these cases the assembly does not take into account previous cytogenetic results, in which chromosome numbering were based for instance on chromosome morphology, banding, gene content, or homology with other species, and not just on the size. Moreover, usually no information is provided on the centromere position, and the p and q arms of a chromosome are not identified. Therefore, a correct direction read of the assembly from p terminus to q terminus is not guaranteed.

This lack of coordination between assembly and previous cytogenetic results leads to a loss of information about genome organization and evolution, and it may generate misunderstandings among investigators. Lewin et al. [18] even proposed a new range of terms for genome assembly elements (i.e., contigs and scaffolds) for those assemblies that are not linked to cytogenetic data. The proposed terminology would distinguish between assemblies that are only scaffold-based from those based on comprehensive physical assignments to chromosomes.

Bridging the gap between genomic and cytogenetic data is the main aim of chromosomics. Originally this term referred to the study of plasticity of chromosomes in relation to the three-dimensional position of genes [19], but today chromosomics denotes an approach that combines cytogenetic with genomic data [20,21,22,23]. Various approaches have been used to link sequencing data with karyotype information, all of them mainly based on Fluorescence in situ Hybridization (FISH). A widely used approach is based on the cloning of genome fragments in Bacterial Artificial Chromosomes (BACs). BAC clones are then sequenced and hybridized on the target species metaphases. FISH results, coupled with the BAC sequence information, give the locations of the sequences on the chromosomes, allowing the construction of a chromosomal map (e.g., [24,25,26,27]). Other approaches are based on the research of previously mapped DNA marker sequences along complete assemblies. If the DNA marker is included in a specific assembled scaffold, this scaffold corresponds to the chromosome where the DNA marker was mapped (e.g., [28]). Such approaches are laborious and time-consuming, and the genome assignment is limited by the number of BACs/markers that can be used in one experiment [29].

Parallel sequencing of DNA from single isolated chromosomes is an elegant approach to determine the chromosome content and directly assign genome scaffolds to chromosomes. The method is based on next generation sequencing of DNA from microdissected or flow-sorted chromosomes. It has been widely employed in plants for the chromosome assignment of assembled genomes, leading to highly significant results (e.g., [30,31,32,33,34,35,36,37]). However, this approach is only now being established in animal genome studies.

The aim of this paper is to describe how single-chromosome sequencing can help in the study of vertebrate genome evolution. We illustrate the role that this approach is playing to bridge the gap between cytogenetic and genomic data in vertebrates, and provide a general overview of the method from both a wet lab and bioinformatic perspective. Finally, we give an example of genome refinement by applying this method to the green anole *Anolis carolinensis*. Single-chromosome sequencing has been previously referred to as ChromSeq in plant genome studies [35] and we will adopt this term throughout the manuscript.

## 2. ChromSeq Workflow

**Figure 1:**
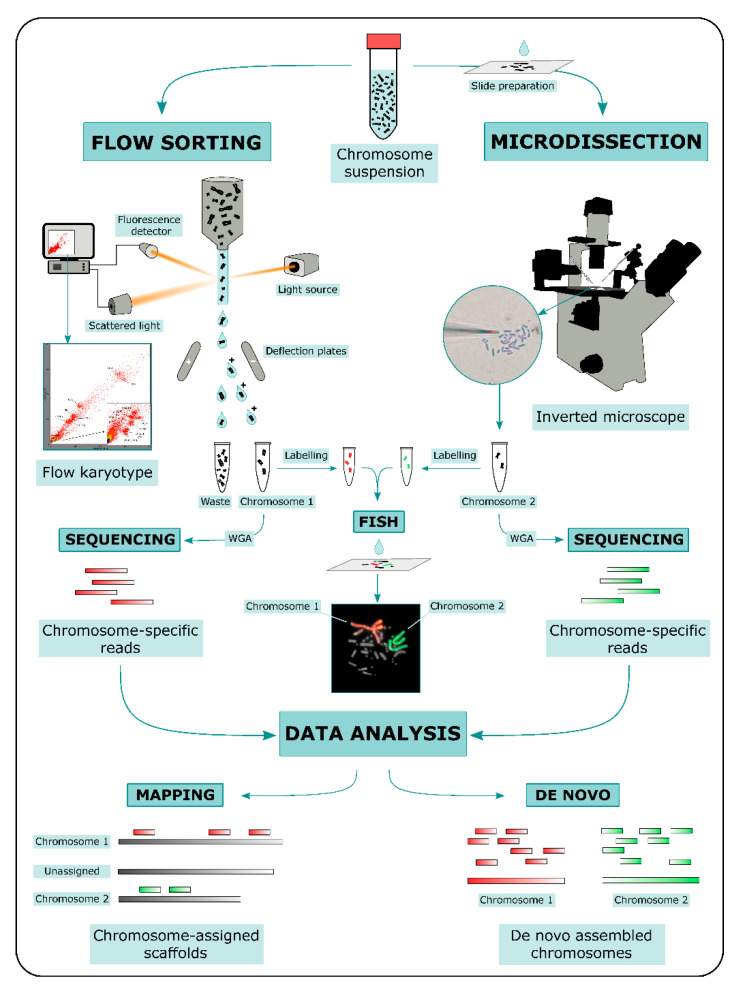
Schematic representation of ChromSeq workflow. Briefly, chromosomes are isolated via either flow sorting or microdissection (only mechanical microdissection is shown). After isolation, Whole-Genome Amplification (WGA) is performed on chromosomal DNA. Eventually, chromosomal DNA can be labeled with fluorochromes and hybridized onto the target species metaphases to confirm the identity of isolated chromosomes. WGA products are then sequenced with next generation sequencing technologies. Sequencing data can be mapped on the target species reference genome or assembled *de novo*. The latter approach has proven successful when a combination of high throughput chromosome isolation (millions of copies) and long-read sequencing approaches are implemented.

ChromSeq workflow consists in three main steps: (i) physical chromosome isolation; (ii) high throughput sequencing of isolated chromosomal DNA; and (iii) bioinformatic analysis of sequencing data (Figure 1).

Two main methods for physical chromosome isolation are currently available: flow sorting and microdissection [38]. Both approaches require the preparation of a metaphase chromosome suspension. Other methods based on microfluidic mechanics have been developed in the last decade for chromosome isolation (e.g., [39,40,41]); however, these methods were not widely used compared to flow sorting and microdissection.

In flow sorting, chromosomal DNA is labeled with two different fluorochromes specific for GC- and AT-rich regions. Fluorochrome-labeled chromosomes are passed through a narrow stream of liquid and broken into fine droplets. Fluorescence intensity is measured for each chromosome contained in a droplet, and the measurements of fluorescence intensities are visualized as a flow karyotype. Ideally, each chromosome forms a distinct peak in the flow karyotype, whose location is proportional to the ratio of GC/AT fluorescence intensity, a relative measure of chromosome size. Peaks can be gated for a specific fluorescence intensity ratio and droplets containing single chromosomes are deflected with an electromagnetic field into tubes [32,42,43]. An advantage of this method is the possibility to isolate a high number of specific chromosomes. However, key disadvantages of the method are the difficulty to separate chromosomes with similar size, and impurity due to the fragmentation of chromosomes.

In microdissection, metaphase spreads on slides are used. Metaphases are observed under an inverted microscope and single chromosomes are physically isolated either with a micromanipulator armed with thin glass needles (mechanical microdissection) or cut out with a laser beam (laser microdissection). In laser microdissection slides are covered with specific membranes to allow the chromosome cut [38,44]. This method generates relatively contamination-free samples and can be used to isolate not only a whole chromosome, but also specific target regions [45,46]. However, microdissection is labor intensive and is restricted to the isolation of usually no more than a dozen chromosomes per type. Moreover, part of the chromosomal DNA can be damaged or lost during the isolation.

Both flow sorting and microdissection yield DNA quantities, which are by themselves too low for high-throughput sequencing. For this reason Whole-Genome Amplification (WGA) is performed on chromosomal DNA prior to sequencing using either degenerate primer (DOP-PCR, Ref. [47]) or multiple displacement amplification (MDA, Refs. [48,49]). An aliquot of the amplified DNA can be used to produce chromosome paints [50]. For this purpose, amplified DNA is labeled with fluorochromes and eventually hybridized onto the target species metaphases, in order to confirm the identity of isolated chromosomes (e.g., [51]). Once a clear correspondence between the isolated chromosomal DNA and the species karyotype is obtained through FISH of chromosome paints, amplified DNA can be used to prepare libraries for high-throughput sequencing according to the manufacturer’s protocols. Currently a short-read sequencing approach is mainly preferred for ChromSeq (e.g., [52,53,54,55,56]), but long-read approaches have also been employed (e.g., [57]).

Sequencing data generated from isolated chromosomes can be processed using a wide variety of approaches that can be divided into two main categories: (i) alignment to a reference genome and (ii) *de novo* assembly of chromosome-specific sequencing data. In cases DOP-PCR or MDA are used prior to the chromosome-specific library sequencing, pre-processing of sequencing data is needed to trim primers and/or adapters independently from the approach used.

Reference-based analysis consists in the alignment of chromosome-specific reads to a reference genome, and represents the most commonly used approach so far. Based on the alignment data, reference genome scaffolds are assigned to specific chromosomes, that is, if reads obtained from chromosome 1 map onto three different scaffolds of the reference genome, it means that those three scaffolds are parts of chromosome 1. If no rearrangement is expected between the reference genome and the sampled chromosome, any statistic for mapped read density can be used to rank scaffolds and subsequently retain those assigned to the chromosome. The problem is further complicated if rearrangements between the target species chromosome and reference genome are possible. In order to predict the rearrangement breakpoints, several methods were successfully developed based on various statistical approaches and read density metrics, including maximum likelihood based on read count per Kb [29,58], circular binary segmentation [56] or clustering [59] based on distances between non-overlapping read mappings. The software DOPseq is the only one developed ad hoc for ChromSeq data analysis and it unifies the chromosome region detection with the upstream processing into an automated and reproducible pipeline [56]. The main disadvantages of a reference-based approach involve errors in read mapping and sample contamination, which can lead to misinterpretation of alignment data. Therefore, it is crucial to separate the true chromosome assignment signal from background noise.

A *de novo* assembly approach can also be implemented on ChromSeq data. In this case, chromosome-specific assemblies are produced independently for each chromosome. This approach requires cross-contamination checks among all chromosome-specific data pools and repetitive sequence removal to increase the assembly contiguity (e.g., [60]). Sequencing data derived from only a few isolated chromosomes are usually highly fragmented and a *de novo* approach might produce assembly with a low contig N50 (e.g., [61]). However, this problem can be circumvented by either sequencing a very large number of chromosome copies (up to millions) or by implementing sequencing data with long-read approaches. Kuderna et al. [62] for instance, successfully assembled *de novo* the human chromosome 1 by using a combination of high throughput chromosome isolation (10 million copies) and Oxford Nanopore sequencing. The resulting assembly had an N50 of 10.5 Mb and allowed the identification of structural variants. The gorilla Y chromosome was also successfully assembled *de novo* using a combination of short and long-read sequencing [57].

## 3. Application in Vertebrate Genome Projects

ChromSeq has been efficiently used in vertebrate genome projects to perform chromosome assignment of assemblies. In this case, chromosome-specific reads mapped onto the assembly are used to assign scaffolds to specific chromosomes and thus link sequence data to cytogenetic data.

ChromSeq was first used in vertebrates to refine the Tasmanian devil (*Sarcophilus harrisii*) genome assembly [63]. The seven Tasmanian devil chromosomes were flow-sorted, and several hundred copies of each devil chromosome were collected, amplified, and sequenced with a short-read approach. Alignment of the chromosome reads with the assembled contigs was used to assign the contigs to chromosomes, and detect and correct assembly errors by identifying contigs with homology to more than one chromosome. ChromSeq allowed assignment of 35,534 supercontigs (99%) to chromosomes [63].

The genome assembly project of the Chinese hamster ovary cell line was also performed based on sequencing of flow-sorted chromosomes [60]. For each flow-sorted chromosome pool, libraries were constructed with Illumina TruSeq protocol, sequenced, and assembled with ALLPATHS-LG. Scaffolds were aligned to the mouse genome, and revealed complex rearrangements and alignment gaps in repeat-rich regions [60].

The genomes of two reptile species, the Komodo dragon (*Varanus komodoensis*) and the green anole lizard (*Anolis carolinensis*), were also significantly improved using ChromSeq. The Komodo dragon genome scaffolds were assigned to chromosomes using sequencing data of each of the 40 chromosomes of the species, which were isolated using flow sorting [64]. This approach led to the assignment of 75% of the whole genome, which represents one of the highest score for reptiles [54]. Similarly, *A. carolinensis* microchromosome scaffold content was revealed through sequencing of flow-sorted chromosomes [53]. This approach enriched the previous assembly produced for the green anole where microchromosomal scaffolds were left unassigned [65]. DOPseq pipelines were used to map chromosome specific reads onto the genome assembly for both the Komodo dragon and green anole lizard.

Recently, ChromSeq has been applied for final genome assembly and validation of chromosome-wide scaffold contents of the sterlet, *Acipenser ruthenus.* Sequencing data derived from individual chromosomes or chromosome arms aligned specifically to individual scaffolds, which were then assigned to either of the homologous sterlet chromosome segments [66].

ChromSeq was also used to assemble and refine limited portions of genome assemblies such as sex chromosomes or specific target regions. For the gorilla Y chromosome, several copies of the target chromosome were isolated through flow sorting and sequenced [57]. The resulting assembly allowed authors to refine gene content, evaluate copy number of ampliconic gene families, locate species-specific palindromes, examine the repetitive element content, and produce sequence alignments with human and chimpanzee Y chromosomes. Sequencing of the laser-microdissected short arm of the frog *Xenopus tropicalis* chromosome 7 allowed the assignment of 200 previously unplaced scaffolds. This chromosome arm is of particular interest as it encodes the sex determination locus [67]. The ChromSeq approach resolved the large gaps contained in previous genetic map [28]. The sex chromosome gene content of another frog species, *Amolops mantzorum*, was also resolved using the same method [61]. ChromSeq was also used to characterize the W chromosome of the flour moth, *Ephestia kuehniella* [68]. Up to now, this is the only example of ChromSeq application in invertebrate species.

Target genomic regions resolved through ChromSeq are not limited to sex chromosomes. A ChromSeq approach was used to link the Japanese eel (*Anguilla japonica*) linkage group 1 (LG1) to cytogenetic data. The species chromosomes were flow-sorted and screened by PCR with primers for a known LG1-linked scaffold. Positive samples were then sequenced and cytogenetically assigned to chromosome 5 by FISH [69].

ChromSeq also refined the sequencing of *Mus musculus* chromosome 17. *De novo* assembly of chromosome 17 was performed using de Bruijn graph-based programs FUZZYPATH and AbySS, leading to the discovery of several regions absent in the mouse reference genome, with a total size of 144 Kb [70].

An improvement of the Asian seabass (*Lates calcarifer*) assembly was also accomplished using ChromSeq. The B chromosomes of this species were microdissected, sequenced and mapped onto the assembled reference Asian seabass genome to build B chromosome pseudo-scaffolds, which were finally assembled using CAP3 [71].

These examples show how ChromSeq has so far been mainly used for the refinement of already assembled genomes. Recently, however, the sequencing of isolated chromosomes was also applied to *de novo* genome assembly of those species that possess particularly difficult-to-assemble genome architectures, including species with large genome sizes (>10 Gb), polyploidy or extensive regions of repetitive elements. Here, ChromSeq enables researchers to partition the genome into several packs of data that can be processed individually, simplifying the assembly task. This approach was widely used in plants as many species are polyploid and possess genomes with high proportion of repeats, which makes genome assembly very challenging. Sequence information of individual chromosomes coupled with genome maps proved to be very useful for genome projects in this taxon (e.g., [31,35,72]).

For vertebrates and animals in general, there is only one notable example of *de novo* genome assemblies based on a ChromSeq approach. In the Axolotl *Ambystoma mexicanum* with a genome size of approximately 32 Gb, Keinath et al. [73] isolated the two smallest *A. mexicanum* chromosomes through laser microdissection, and amplified and sequenced chromosomal DNA. Chromosome-targeted sequencing allowed the development of an initial assembly within the constraints of modern computational platforms and enabled authors to place 2062 genes on the two smallest *A. mexicanum* chromosomes [73]. Altogether these data laid the foundation for production of a complete genome assembly of the Axolotl [74]. The genome of the Apennine yellow-bellied toad *Bombina pachypus* is also being assembled *de novo* using a ChromSeq approach, given its relatively large genome size of 10 Gb.

## 4. Application in Vertebrate Karyotype Evolution Studies

Apart from being an efficient tool to improve genome assembly resolution, ChromSeq was also used for the study of vertebrate comparative genomics and karyotype evolution. Various approaches have been implemented to map homologies between chromosomes and chromosome fragments of different species and to reconstruct the evolution of their karyotypes. Conventional cytogenetic methods take into account chromosome sizes, morphologies and differential staining. However, these methods have limited applicability if the karyotypes of the studied species have accumulated many rearrangements. In the case of extremely high rates of chromosome evolution visually recognizable syntenic fragments of a given size, morphology and banding pattern may be very difficult to recognize. More recent molecular cytogenetic developments, such as FISH with probes for individual genes, and with whole-chromosome paints, overcame these limitations and proved to be extremely efficient for the study of karyotype evolution in many vertebrate lineages (e.g., [75,76,77,78,79,80,81]). However, FISH may still fail to detect fine scale chromosome rearrangements, and, most importantly, FISH alone does not allow an investigation of chromosome evolution at the sequence-level resolution.

ChromSeq was successfully utilized to elucidate chromosomal evolution of many vertebrate lineages. In fish, a ChromSeq approach was implemented to study the evolution of the sterlet (*Acipenser ruthenus*) karyotype [52]. This is a paleotetraploid species, which experienced several inter-chromosomal rearrangements after the tetraploidization event. Using sequencing data of the microdissected whole-chromosome libraries, rearrangements were identified and it was shown that different chromosomes were unequally involved in this process [52].

The study of sex chromosome evolution—and neo-sex chromosomes in particular—has also taken great advantage of the ChromSeq approach. This technique was implemented to reveal the genetic contents of neo-sex chromosomes in two iguanian lizard groups, which independently experienced multiple whole-chromosome fusions: the anoles (Dactyloidae) and the fence lizards (*Sceloporus*, Phrynosomatidae) [55,82]. Despite the evolutionary independence of the fusions, repeated fusions of the same ancestral chromosomes were identified, which suggests that such fusions may probably occur non-randomly or may have non-neutral consequences and be fixed by selection.

Kichigin et al. [53] also used ChromSeq to investigate karyotype evolution and evolutionary dynamics of sex chromosomes in the genus *Anolis*. The method allowed for the comparison of sex chromosomes at a sequence level revealing that the *A. sagrei* XY sex chromosomes contain regions homologous to several micro autosomes of *A. carolinensis*. This led to the conclusion that sex chromosomes of *A. sagrei* are probably derived by fusions of the ancestral sex chromosome with three micro autosomes followed by a subsequent loss of some genetic content on the Y chromosome.

ChromSeq has also proven to be an efficient method to study the evolution and genetic contents of supernumerary chromosomes. This approach was first developed by Makunin et al. [56], who studied the B chromosomes of two deer species, the Siberian roe deer (*Capreolus pygargus*) and the grey brocket deer (*Mazama gouazoubira*). It was found that these chromosomes drastically differed in size (1.42–1.98 Mb and 8.28–9.31 Mb, respectively) and in genetic content. These results suggested that they had independent origins. In the B chromosomes of the brocket deer, two proto-oncogenes were found: *KIT* and *RET*. The same genes had previously been found in the B chromosomes of canids, suggesting that genomic regions involved in B chromosome formation in different species are not random [56]. The ChromSeq analyses of B chromosomes from other species, such as the field mice (*Apodemus*), *A. carolinensis*, and the cichlid fish *Astatotilapia latifasciata*, revealed similar contents and organization: the B chromosomes are enriched with repeats and with genes related to the cell cycle, and show signs of pseudogenization [83,84,85]. In the Asian seabass (*Lates calcarifer*) ChromSeq was eventually able to reveal that B chromosome diversity correlates with the geographic structure of populations [59].

In the groundbreaking work devoted to the discovery of a germline-restricted chromosome (GRC) in songbirds, ChromSeq was used to determine the genetic contents of the supernumerary GRCs [86]. Despite the single evolutionary origin of the GRC, as shown by phylogenetic analysis, its sequence content differed drastically between species. This showed that dissimilarity in genetic content does not necessarily mean that supernumerary chromosomes in different species have independent origins. Interestingly, the efficiency of ChromSeq in this work was further supported by the fact that ChromSeq and FISH with the GRC probes revealed the same segments of A-chromosomes, from which parts of the GRC genetic contents were derived. It is therefore evident that ChromSeq, combined with FISH, is a very promising innovative technique, applicable in many areas of evolutionary cytogenetic research.

## 5. ChromSeq to Refine *Anolis carolinensis* Genome Assembly

To provide an example of how ChromSeq can support genome assembly refinement, we analyzed sequencing data of *Anolis carolinensis* chromosome 6 (ACA6). This chromosome was sequenced as part of the project published by Kichigin et al. [53] on *Anolis* sex chromosomes (see above).

We reanalyzed the data of chromosome 6 with DOPseq v.2.1.1 (https://github.com/lca-imcb/dopseq). Out of 352,644 reads generated on Illumina MiSeq, 245,090 were mapped to the reference genome of *A. carolinensis* (AnoCar2.0, NCBI accession numbers: PRJNA18787) with BWA-MEM v.0.7.15 [87]. PCR duplicate removal with Picard v.2.9.2 resulted in the removal of 70% reads, and after further filtering (alignment length 20, MAPQ 20), 57,191 reads were retained with a 2% average divergence from the reference (SNPs and indels combined). Merging of overlapping mapped reads resulted in 20,957 distinct positions. Of those, 9293 (44%) were located on chromosome 6 of AnoCar2.0, as expected.

An additional region was detected on AnoCar2.0 chromosome 1 starting at 192,636,407 bp and ending at 196,507,123 bp. ChromSeq data from ACA6 mapped in 424 positions within this region (one position every 4.7 Kb, Figure S1). This region may represent either a translocation in the sampled genome or a misassembly of AnoCar2.0. Two factors favor the latter explanation. Firstly, there is a correspondence of the region margins with the end of AnoCar2.0 contigs belonging to that region, confirming a discontinuity of the assembly. Secondly, the homology between this region and that of other species is different if compared to the homology between the flanking regions and those of the same species. For instance, this region is homologous to *Gallus gallus* chromosome 2 (GGA2) and *Homo sapiens* chromosome 3 (HSA3), while the flanking regions are homologous to GGA3 and HSA6.

Besides the discovery of a possible misassembly, our analysis allowed to assign a set of at least 34 scaffolds that were left unassigned in AnoCar2.0. These scaffolds can be attributed to ACA6 based on stringent criteria (at least 15 positions, enrichment *p*-value < 0.05). They constitute a total of 28.9 Mb and the individual scaffold sizes range from 140 to 2530 Kb (Table S1).

The example of ACA6 shows how ChromSeq can be efficiently used to refine vertebrate genome assemblies and eventually correct genome misassemblies.

## 6. Conclusions

In the last decades, genomics has proven to be fundamental in the study of vertebrate genome evolution. An impressive and admirable effort is currently underway to generate high-quality, complete reference genomes for all ~70,000 extant vertebrate species to enable a new era of discovery across this field [17]. Before the advent of genomics, information on vertebrate genome evolution was mainly gained through cytogenetic approaches that relied on chromosome morphology, banding patterns, and FISH experiments (see [88] for a comprehensive review on vertebrate chromosome evolution). Linking cytogenetic data with novel genomic discoveries is a worthy departure point for future research. In this paper, we described how ChromSeq can create a link between traditional cytogenetic approaches and sequence assemblies. We also illustrated how the sequencing of single chromosomes can support genome refinement to provide a bridge between cytogenetics and genomics as needed.

Future perspectives in this field should aim at improving the sequencing approaches of single chromosomes to reach higher resolution data. A combination of novel long-read technologies together with the development of new bioinformatic tools for the *de novo* assembly of single chromosomes data will certainly consolidate the field of chromosomics and will continue to strengthen the bridge between cytogenetic and genomics.

## Data Availability

Raw sequences of *Anolis carolinensis* chromosome 6-specific DNA pool are available at NCBI sequence read archive (SRA), under the accession number SRR3223634.

## Supplementary Materials

The following are available online at https://www.mdpi.com/2073-4425/12/1/124/s1, Figure S1: Graphic representation of AnoCar2.0 chromosome 1 assignment based on ACA6 ChromSeq data. Table S1: AnoCar2.0 genome assignment results based on ACA6 ChromSeq data.
