# Supplementary material for: Bridging the Gap between Vertebrate Cytogenetics and Genomics with Single-Chromosome Sequencing (ChromSeq)
Source: Genes (Basel). 2021 Jan 19;12(1):124. doi: 10.3390/genes12010124 (PMC7835784; doi:10.3390/genes12010124)
Supplement: Supplementary file 1 [file genes-12-00124-s001.zip › Iannucci et al. - Supplementary Materials.docx]

**Supplemental Information for:**

**Bridging the gap between vertebrate cytogenetics and genomics with single-chromosome sequencing (ChromSeq)**

Alessio Iannucci *^1^, Alexey I. Makunin ^2,3^, Artem P. Lisachov ^4,5^, Claudio Ciofi ^1^, Roscoe Stanyon ^1^, Marta Svartman ^6^, Vladimir A. Trifonov ^3^

^1^ Department of Biology, University of Florence, 50019 Sesto Fiorentino (FI), Italy

^2^ Wellcome Sanger Institute, Hinxton, Cambridgeshire, CB10 1SA, UK

^3^ Institute of Molecular and Cellular Biology SB RAS, 630090 Novosibirsk, Russia

^4^ Institute of Environmental and Agricultural Biology (X-BIO), University of Tyumen, 625003 Tyumen, Russia

^5^ Institute of Cytology and Genetics SB RAS, 630090 Novosibirsk, Russia

^6^ Departamento de Genética, Ecologia e Evolução, Universidade Federal de Minas Gerais, CEP 31270-901, Belo Horizonte/MG, Brazil

**Correspondence:** Alessio Iannucci, Department of Biology, University of Florence, 50019 Sesto Fiorentino (FI), Italy. Email: [alessio.iannucci@unifi.it](mailto:alessio.iannucci@unifi.it)


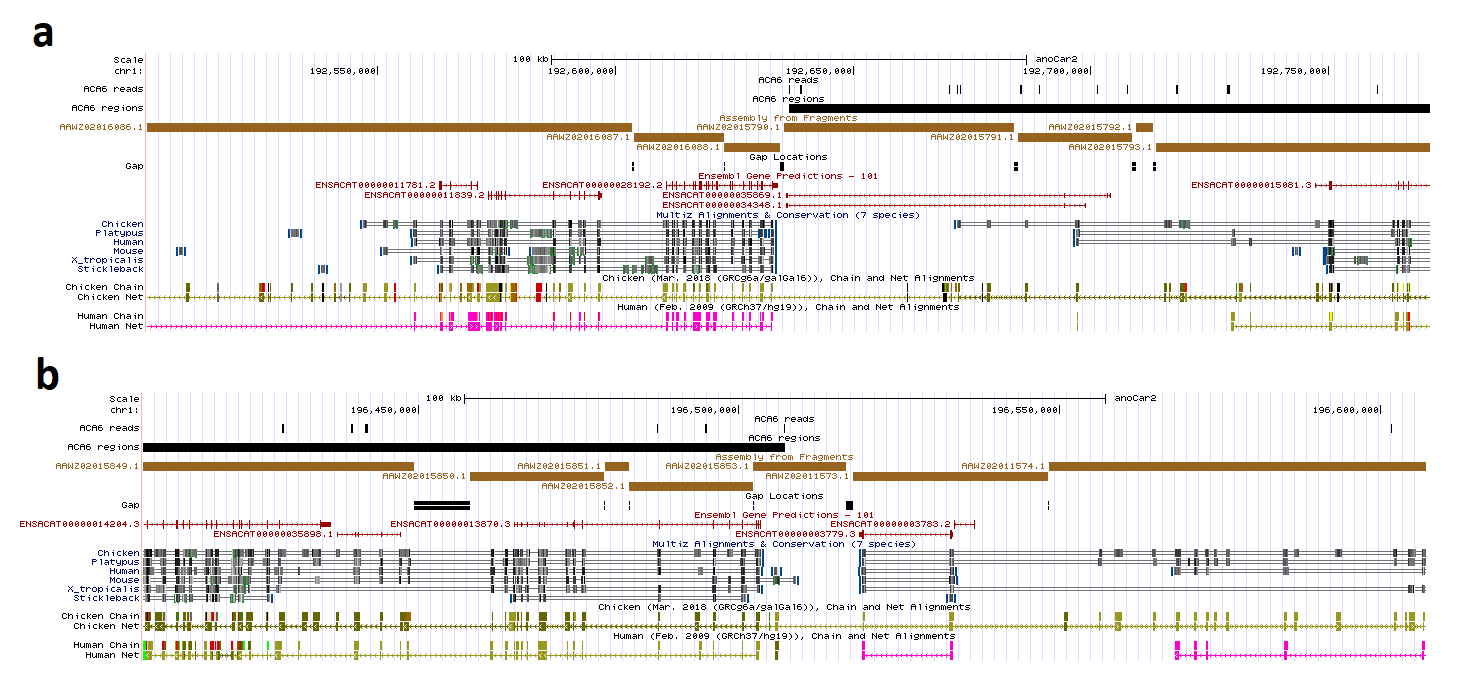


**Figure S1.** Portion of chromosome 1 of *Anolis carolinensis* genome AnoCar2.0 (brown). Based on ACA6 ChromSeq results, a region of this portion is assigned to *A. carolinensis* chromosome 6 (black). This region may represent a misassembly of AnoCar2.0. In fact, there is a correspondence of the left (a) and right (b) margin of the region with the end of AnoCar2.0 contigs belonging to that region. Moreover, the homology between this region and that of other species is different if compared to the homology between the flanking regions and those of the same species.
